# Intranasal insulin attenuates hypoxia-ischemia-induced short-term sensorimotor behavioral disturbances, neuronal apoptosis, and brain damage in neonatal rats

**DOI:** 10.1016/j.crneur.2023.100123

**Published:** 2023-12-27

**Authors:** Chirag P. Talati, Jonathan W. Lee, Silu Lu, Norma B. Ojeda, Varsha Prakash, Nilesh Dankhara, Tanner C. Nielson, Sara P. Sandifer, Gene L. Bidwell, Yi Pang, Lir-Wan Fan, Abhay J. Bhatt

**Affiliations:** a Department of Pediatrics, Division of Newborn Medicine, University of Mississippi Medical Center, Jackson, MS, 39216, USA; b Department of Neurology, University of Mississippi Medical Center, Jackson, MS, 39216, USA; c Department of Pathology, University of Mississippi Medical Center, Jackson, MS, 39216, USA

**Keywords:** Hypoxia-ischemia, Intranasal insulin, Neuron apoptosis, Sensorimotor dysfunction, Neuroprotection

## Abstract

There is a significant need for additional therapy to improve outcomes for newborns with acute Hypoxic-ischemic (HI) encephalopathy (HIE). New evidence suggests that insulin could be neuroprotective. This study aimed to investigate whether intranasal insulin attenuates HI-induced brain damage and neurobehavioral dysfunction in neonatal rats. Postnatal day 10 (P10), Sprague-Dawley rat pups were randomly divided into Sham + Vehicle, Sham + Insulin, HI + Vehicle, and HI + Insulin groups with equal male-to-female ratios. Pups either had HI by permanent ligation of the right common carotid artery followed by 90 min of hypoxia (8% O2) or sham surgery followed by room air exposure. Immediately after HI or Sham, pups were given fluorescence-tagged insulin (Alex-546-insulin)/vehicle, human insulin (25 μg), or vehicle in each nare under anesthesia. Shortly after administration, widespread Alex-546-insulin-binding cells were detected in the brain, primarily co-localized with neuronal nuclei-positive neurons on double-immunostaining. In the hippocampus, phospho-Akt was activated in a subset of Alex-546-insulin double-labeled cells, suggesting activation of the Akt/PI3K pathway in these neurons. Intranasal insulin (InInsulin) reduced HI-induced sensorimotor behavioral disturbances at P11. InInsulin prevented HI-induced increased Fluoro-Jade C+ degenerated neurons, cleaved caspase 3+ neurons, and volume loss in the ipsilateral brain at P11. There was no sex-specific response to HI or insulin. The findings confirm that intranasal insulin provides neuroprotection against HI brain injury in P10 rats associated with activation of intracellular cell survival signaling. If further pre-clinical research shows long-term benefits, intranasal insulin has the potential to be a promising non-invasive therapy to improve outcomes for newborns with HIE.

## Highlights

• Insulin on intranasal instillation following hypoxic-ischemic exposure in P10 rats.
  o quickly penetrates and distributes in brain without causing hypoglycemia
  o reduces HI-induced short-term neurobehavioral deficits and ipsilateral brain damage
  o has similar effects in male and female
• Proof of concept study for a novel non-invasive therapy for newborns with HIE

## Graphical abstract

**Figure:**
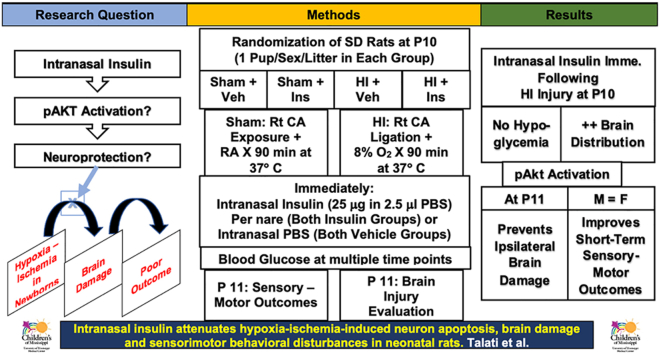

## 1 Introduction

Despite current advances in neonatal care and a newer therapy of moderate therapeutic hypothermia (TH), hypoxic-ischemic (HI) encephalopathy (HIE) still causes significant morbidity and mortality in the neonatal period (Shankaran et al., 2017). TH induced by cooling either the head or the whole body is the only treatment currently employed to reduce death and disability, and only in late preterm and term infants (Shankaran et al., 2005); currently, the benefits of initiation of TH beyond 6 h of an HI insult is uncertain (Laptook et al., 2017). Furthermore, recent randomized control trials and review articles state that the incidence of death and disability remains high despite treatment with cooling (21- 40%) (Laptook et al., 2017; Shankaran et al., 2005), pointing to an urgent need for additional therapies to further improve outcomes of infants who have acute HIE (Committee on et al., 2014).

Brain injury following HI is an evolving process initiated during the insult and extends into a recovery period, the “reperfusion phase,” which is amenable to potential intervention (Hagberg et al., 2016; Vannucci and Perlman, 1997). Since the brain's energy metabolism is independent of insulin, and insulin receptors are expressed by neural cells, it has been proposed that insulin might play yet unknown physiological roles in the central nervous system (Banks et al., 2012). Recent animal and clinical studies suggest that insulin could function as a neuroprotective agent through the PI3K (phosphoinositide 3-kinase) pathway (also known as protein kinase B, a serine/threonine-protein kinase) and its resulting activation of phosphorylated AKT (p-AKT) (Kim and Han, 2005; Ribeiro et al., 2014; Sun et al., 2010). Insulin modulates higher brain functions, including cognition and appetite. Intranasal insulin (InInsulin) could enhance long-term declarative memory in healthy human volunteers (Benedict et al., 2004; Reger et al., 2006), as well as improve verbal memory in Alzheimer's patients (Dubey et al., 2020; Reger et al., 2008). Insulin is closely related to insulin-like growth factor-1 (IGF-1), a well-known growth and trophic factor. In the brain, IGF-1 plays an important role in neurodevelopment as well as in neuroprotection against perinatal HI (Fernandez and Torres-Aleman, 2012). Since both IR and IGF-1 receptors (IGF-1R) are widely expressed in the brain, and their intracellular signaling pathways largely overlap, insulin could function as a neuroprotective agent once it reaches the brain parenchyma at sufficient levels. In vitro studies have shown that insulin protects neurons against a variety of insults, including glucose-oxygen deprivation (Sun et al., 2010), excitotoxicity (Kim and Han, 2005), and oxidative stress (Ribeiro et al., 2014). The neuroprotective property of insulin has not been investigated in an in vivo model of HI. A growing body of evidence from rodent models and human studies supports the intranasal route for direct nose-to-brain delivery of drugs for various neurological disorders that bypass the blood-brain barrier altogether (Crowe and Hsu, 2022). In human studies, insulin is detectable in the cerebrospinal fluid within 30 min after nasal administration (Freiherr et al., 2013), suggesting that intranasal administration may be a practical means to deliver insulin into the brain parenchyma.

To evaluate the prospect of InInsulin as a neuroprotective agent, we hypothesized that InInsulin provides neuroprotection against short-term adverse outcomes following neonatal HI. We investigated our hypothesis in a 10-day-old rat model of HI-induced by the method of Levine with modification by Rice et al. (1981), Lin et al. (2011), and Patel et al. (2015). Our objectives were to investigate whether InInsulin mitigates neuronal apoptosis, short-term microscopic changes of brain injury, and neurobehavioral outcomes following HI in newborn rats. In addition, we also evaluated the sex-specific effects of HI and InInsulin.

## 2 Materials and methods

### 2.1 Chemicals

Unless otherwise stated, all chemicals used in this study were purchased from Sigma (St. Louis, MO, USA). Recombinant human insulin (rh-Insulin) was purchased from Cell Sciences (Newburyport, MA, USA). Alexa Fluor 546-labeled insulin (Alex-546-Insulin) was purchased from Nanocs Inc (Farmingdale, NY, USA). Monoclonal mouse antibodies against the neuron-specific nuclear protein (NeuN) and Fluoro-Jade C were purchased from Millipore (Billerica, MA, USA). Polyclonal rabbit antibodies against phosphor-Akt (pAkt) and cleaved caspase 3 (Cl-Caspase 3) were obtained from Cell Signaling Technology (Danvers, MA, USA). ELISA kits for immunoassays of human insulin were purchased from Alpco (Salem, NH, USA).

### 2.2 Animals

Pregnant Sprague-Dawley rats arrived at the animal care facility on day 19 of gestation. Animals were maintained in an animal room on a 12-hr light/dark cycle at constant temperature (22 ± 2 °C). The day of birth was defined as postnatal day 0 (P0). After birth, the litter size was adjusted to 10 pups per litter to minimize the effect of litter size on body weight and brain size. All procedures for animal care were conducted in accordance with the National Institutes of Health Guide for the Care and Use of Laboratory Animals, and were approved by the Institutional Animal Care and Use Committee at the University of Mississippi Medical Center. Every effort was made to minimize the number of animals used and their suffering.

### 2.3 Surgery procedures and animal treatment

The surgery procedures were performed as previously described with modification (Lin et al., 2011; Patel et al., 2015; Rice et al., 1981). The operation was performed on 10-day-old (P10) Sprague-Dawley rats of both sexes. Pups from each litter were divided by sex and assigned numbers. Pups were randomly divided into four groups: Sham + Vehicle, Sham + Insulin, HI + Vehicle, and HI + Insulin. The male and female ratio was kept equal in each group. Each litter contributed at most 1 male and 1 female pup in any group. Under light anesthesia with isoflurane (1.5∼5%), right common carotid artery ligation was performed with 4–0 silk sutures under a surgical microscope. For the sham group, the right common carotid artery was exposed but not ligated. After the wound was sutured, animals were placed on a warm heating pad (37 °C) for recovery from anesthesia. All animals survived the operation. After 2 h recovery with their dams, rat pups were exposed to a hypoxic condition (a humidified gas mixture of 8% oxygen with balanced nitrogen delivered at a rate of 4 L/min) for 90 min. During the hypoxic exposure, rat pups were placed in a 1-L sealed glass jar, which was partially submerged in a 37 °C water bath to maintain a constant thermal environment. For the sham group, pups were separated from dams and exposed to room air in a 37 °C water bath for 90 min. Pups were then returned to their dams and allowed to recover and grow for follow-up experiments. Pups in the insulin groups were held upright while under light anesthesia with isoflurane, and 25 μg of rh-insulin or fluorescence-tagged insulin (Alex-546-insulin) (in 2.5 μl PBS) was applied to each naris using a 5 μl fine pipette tip. Each rat received a total of 50 μg of insulin (1.44 units of insulin). Pups in the Vehicle groups received the same amount of sterile PBS under light anesthesia. The dose of 50 μg was selected by extrapolating, based on average weight, the effective dose as derived by Pang et al. studying intranasal insulin's effects on 6-OHDA induced brain injury in rats (Fan et al., 2019; Pang et al., 2016).

Each dam had the same litter size (10 pups), and equal numbers of rat pups (16 male and 16 female pups) for each treatment group were taken from sixteen different litters.

The remaining pups were used for other experiments. Eight rat pups (4 males and 4 females) from each group were sacrificed by decapitation 15 min after the treatment for IVIS imaging, and another set of pups (4 males and 4 females for each group) were used for the collection of fresh brain tissues. One day after the operation (P11), 16 rat pups (8 males and 8 females) for each group were sacrificed by transcardiac perfusion with normal saline followed by 4% paraformaldehyde under isoflurane anesthesia for brain section preparation.

### 2.4 Blood glucose level measurement

Blood glucose levels were measured using a Contour Blood Glucose Monitoring System (Bayer, Leverkusen, Germany). A small drop of blood was obtained from rat tail veins and applied to a test strip, and the glucose level was read immediately. A baseline level was determined 1 h before surgery. Blood glucose was measured at 0, 30 min, and 24 h after rh-insulin administration. The glucose contents were expressed as milligrams per dl of blood.

### 2.5 IVIS imaging and enzyme-linked immunosorbent assay (ELISA)

To study the distribution of insulin in the brain following intranasal application, one set of pups was administered Alexa-546-labeled insulin (Nanocs Inc, Farmingdale, NY, USA). Fifteen min after Alexa-546-insulin (red color)/Vehicle administration, the pups were anesthetized with an overdose of isoflurane, and their brains and spinal cords were surgically removed. The brains and spinal cords were placed in the chamber for *ex vivo* IVIS imaging (IVIS Spectrum Imager, PerkinElmer Inc., Waltham, MA, USA) (McGowan and Bidwell, 2016). The brains were then placed in 4% paraformaldehyde for brain section preparation. Another set of animals was prepared for the ELISA study. The right and left cerebellum, brain stem, hippocampus, cerebral hemispheres, and spinal cord were dissected on ice, snap-frozen in dry ice, and stored at −80 °C for future processing and analysis. Total proteins were extracted using protein lysis buffer (Millipore, Billerica, MA, USA). An ultrasensitive ELISA kit (ALPCO, Catalog# 80-INSHUU-E0.1) was used to measure tissue insulin levels (the kit is specific for human but not rat insulin). Serum of diabetic patients and rh-insulin (100 pg/ml) were included in each ELISA kit as positive controls. Normal rat brain tissue from corresponding brain regions was run in each ELISA to determine the baseline. The rh-insulin contents were expressed as picograms per mg protein in the brain or spinal cord or picograms per ml of serum.

### 2.6 Behavioral testing

The developmental tests battery used was based on the tests for neurobehavioral injury (Altman and Sudarshan, 1975; El-Khodor et al., 2008; Hermans et al., 1992).

Righting reflex, negative geotaxis, wire hanging maneuver, and hind-limb suspension tests were performed on rat pups at P11 to measure neurological function at the early developmental stage. The behavioral tests were performed by an investigator blind to the treatment as described previously (Fan et al., 2005, 2006; Lan et al., 2016) with modifications. All animals were tested in the same order. The body weights of rats were recorded on P11.

#### 2.6.1 Righting reflex

Righting reflex is used to test for reflection of muscle strength and subcortical maturation (Altman and Sudarshan, 1975; Hermans et al., 1992). P11 pups were placed on their backs, and the time required to turn over on all four feet and touch the platform was measured. Each pup was given three trials, and the time spent to complete one turnover was recorded. The cut-off time was 60 s.

#### 2.6.2 Negative geotaxis

This test is believed to test reflex development, motor skills, vestibular labyrinth, and cerebellar integration (Altman and Sudarshan, 1975; Hermans et al., 1992). P11 pups were placed on a 15° incline with their heads pointing down the slope, and the time required to turn around to face upward and begin to crawl up the slope was measured. Each pup was given three trials, and the time spent for a turn of 180° upward was recorded. The cut-off time was 60 s.

#### 2.6.3 Wire hanging maneuver

This maneuver tests neuromuscular and locomotor development (Altman and Sudarshan, 1975; Hermans et al., 1992). Pups suspended by their forelimbs from a horizontal rod (5 x 5 mm^2^ area, 35 cm long, between two poles 50 cm high) tend to support themselves with their hind limbs, preventing them from falling and aiding in progression along the rod. A sawdust-filled box at the base served as protection for the falling pups. Each pup was given three trials on P11, and suspension latencies were recorded. The cut-off time was 120 s.

#### 2.6.4 Hind-limb suspension test

This test evaluates the proximal hind-limb muscle strength, weakness, and fatigue in rat neonates (El-Khodor et al., 2008). Each pup was given three trials on P11. In each trial, the rat pups were placed head down inside a plastic cylinder (4 cm inside diameter and 16 cm height), suspended by their hind limbs from the lip of the cylinder, with a cotton ball cushion at the bottom to protect the animal's head upon falling. Suspension latencies were recorded, and the cut-off time was 120 s.

### 2.7 Immunohistochemistry, Fluoro-Jade C, and Nissl staining

Brain apoptosis and brain injury were estimated based on the results of immunohistochemistry, Fluoro-Jade C staining, and Nissl staining in consecutive frozen coronal sections at a thickness of 40 μm prepared from the P10 and P11 rat brain (15 min and 1 day after the HI insult).

#### 2.7.1 Immunohistochemistry

For immunohistochemistry, NeuN (1:400) was used to detect the neuron-specific nuclear protein, which is primarily localized in the nucleus of the neurons with slight staining in the cytoplasm. pAkt (1:400) was used to detect phosphorylated Akt, an activated type of Akt by phospholipid binding to promote cell survival by inhibiting apoptosis through phosphorylation and inactivation of several targets. Cl-Caspase-3 (1:300) was used to detect cleaved caspase-3, an activated type of caspase-3 and a critical executioner of apoptosis.

For co-labeling Alex546-Insulin with NeuN and pAkt, triple immunofluorescence staining was performed. First, sections were blocked with 10% normal goat serum and 0.2% Triton X-100 in PBS for 30 min at room temperature and incubated with primary antibodies NeuN and pAkt overnight at 4 °C. The next day, sections were washed with PBS and then incubated with secondary antibodies conjugated with biotin, Alexa Fluor 488 (1:300), or Alexa Fluor 405 (1:300) for 1 h in the dark on a plate shaker at room temperature. Sections were washed in PBS, mounted on slides, and air-dried.

Double immunofluorescence staining was performed with NeuN and Cl-Caspase 3.

Sections were blocked with 10% normal goat serum and 0.2% Triton X-100 in PBS for 30 min at room temperature and incubated with primary antibodies NeuN and pAkt overnight at 4 °C. The next day, sections were washed with PBS and then incubated with secondary antibodies conjugated with biotin, Alexa Fluor 488 (1:300), or Alexa Fluor 555 (1:500) for 1 h in the dark on a plate shaker at room temperature. Sections were washed in PBS, mounted on slides, and air-dried.

4′, 6-Diamidino-2-phenylindole (DAPI) (100 ng/ml) was used simultaneously to counterstain nuclei and aid in identification during the final visualization. Negative control sections were incubated in the absence of a primary antibody. The resulting sections were examined under a fluorescent microscope (Nikon Ni-E, Melville, NY, USA) at appropriate wavelengths.

#### 2.7.2 Fluoro-Jade C staining

HI-induced neuronal death was assessed by Fluoro-Jade C (Millipore, Billerica, MA, USA), a marker of degenerating neurons. It is a polyanionic fluorescein derivative that sensitively and specifically binds to degenerating neurons (Schmued et al., 2005). Brain sections were incubated with 1% NaOH/80% ethanol followed by 0.06% potassium permanganate for 5 and 15 min, respectively, to block the background staining, and then with 0.0002 % Fluoro-Jade C and DAPI (0.0001%, used to counterstain DNA) for 30 min before air-drying in the dark.

#### 2.7.3 Nissl staining

Nissl granules are distributed in the cytoplasm and dendrites of neurons, and Nissl staining is used to examine the cellular patterns of the brain (Zhu et al., 2015). Brain sections were demyelinated through an alcohol series and then incubated in a 0.5% cresyl violet solution for 3 min.

#### 2.7.4 Quantification of staining data

To compare the cell number among the treatment groups, positively stained cells were counted. Three sections at each of the two levels (bregma and middle dorsal hippocampal levels) were examined by an observer blind to the treatment. For each section, three randomly captured digital microscopic images were taken using Stereology System (MBF Bioscience, Williston, VT, USA) at the areas where the positive cells were abundant, mainly the hippocampal area in the ipsilateral and contralateral hemispheres. The number of positively stained cells in the three images was then averaged. The mean value of the three sections from two levels was used to represent one single brain. For the convenience of comparing results among the treatment groups, results were standardized as the average number of pAkt + or Cl-Caspase 3+ cells per mm^2^ in the hippocampal area of the ipsilateral and contralateral hemispheres.

#### 2.7.5 Stereological estimates of the volume of the cerebrum and the total number of Fluoro-Jade C+ cells in the brain

Brain injury was estimated based on the results of Nissl staining on consecutive brain sections prepared from rats sacrificed 1 day (P11) after the treatment. Brain damage was assessed by measuring the decrease in tissue density when magnified or significant changes in parenchymal architecture compared to the contralateral side. The stereological estimates of the total volume of the cerebrum, cortex, striatum, and hippocampus were determined using methods described previously (Lan et al., 2015). The fifty-six equally spaced thick (40 μm) sections used in the analysis came from a one-in-six series. Nissl-stained sections were scanned by a densitometer (Bio-Rad Hercules, CA, USA), and the areas of the cortex, striatum, and hippocampus, as well as that of the whole brain section (cerebrum), were outlined and determined using NIH image software in each of the fifty-six sections (Fan et al., 2011; Lan et al., 2015). The Cavalieri principle (Gundersen and Jensen, 1987) was used to estimate the reference volumes, *est V(ref)*.

The stereological estimates of the total number of Fluoro-Jade C+ cells in the cerebrum were performed in the P11 rat brain following the methods described previously (Fan et al., 2011; Ling et al., 2006; Pang et al., 2016; Tien et al., 2017). The fifty-six equally spaced thick (40 μm) sections used in the analysis came from a one-in-six series. The total number of Fluoro-Jade C+ labeled cells (*est N*) were counted in each of the fifty-six sections.

The Cavalieri principle (Gundersen and Jensen, 1987) was used to estimate the reference volumes, *est V*(*ref*), and the volume density, *est NV*. The product of the two is an estimate of the total number of cells in this region: *est V(ref)* X *est NV = est N* (Ling et al., 2006; Lokkegaard et al., 2001; Pakkenberg and Gundersen, 1988). As described previously (Pang et al., 2016; Tien et al., 2017), the images were taken under unbiased conditions using Stereo Investigator software (MBF Bioscience, Williston, VT, USA) with a motorized microscope (Nikon Ni-E, Melville, NY, USA). Imaged cells were then examined and quantified with the software.

### 2.8 Statistical analysis

Statistical analysis was performed using SigmaPlot Ver 14.0 software. All values from all pups were included in the analysis. Results with a *p<0.05* were considered statistically significant.

Data of rh-insulin levels in ELISA are presented as the mean±SD in Table 1. Statistical analysis was performed the Kruskal-Wallis One Way Analysis of Variance on Ranks followed by the Student-Newman-Keuls test for post-hoc analysis. For the rest of the results, statistical analysis was performed with two-way ANOVA (with one factor being Sham/HI and the second Vehicle/Insulin), followed by Holm-Sidak testing for post-hoc analysis. To evaluate sex-specific effects, three-way ANOVA was used. Male and female pups were examined separately by the Kruskal-Wallis One Way Analysis of Variance on Ranks, followed by the Student-Newman-Keuls test (for equal group sizes) or Dunn test (for unequal group sizes) for post-hoc analysis. Data were presented as median and range by the box and whisker plot; mean value and outliers are also shown.

**Table 1:** Recombinant human insulin concentration (pg/mg of tissue, pg/ml for serum) in different brain regions at 15 min after Intranasal Insulin/Veh administration following HI/Sham exposure in P10 rats.

Source	Sham + Veh	Sham + Veh	HI + Veh	HI + Veh	Sham + Insulin	Sham + Insulin	HI + Insulin	HI + Insulin
	M	F	M	F	M	F	M	F
**Rt OB**	0.5 ± 1.1	0.1 ± 1.7	0.2 ± 2.3	0.2 ± 1.6	45.9 ± 13.5*	38.2 ± 7.7*	47.3 ± 15.2*	16.1 ± 3.3*
**Lt OB**	1.2 ± 1.2	0.6 ± 0.9	1.2 ± 1.0	0.9 ± 1.3	44.6 ± 11.3*^+^	36.2 ± 6.0*^+^	56.7 ± 17.6*^+§^	23.8 ± 3.7*^+ζ^
**Rt FC**	0.1 ± 1.6	0.7 ± 0.7	1.1 ± 1.8	1.8 ± 1.0	17.7 ± 7.7*^+^	17.5 ± 4.9*^+^	12.4 ± 3.2*^+§^	7.4 ± 2.4*^+ζ^
**Lt FC**	0.3 ± 1.7	0.4 ± 1.5	0.5 ± 1.9	1.5 ± 1.0	16.1 ± 3.4*	19.8 ± 9.1*	16.7 ± 9.3*	18.4 ± 5.6*
**Rt ST**	1.4 ± 0.9	0.3 ± 0.6	0.8 ± 1.5	1.0 ± 1.3	16.5 ± 3.8*^+^	17.5 ± 3.6*^+^	14.1 ± 4.8*^+^	8.5 ± 1.1*^+ζ^
**Lt ST**	1.3 ± 0.6	0.9 ± 0.9	0.2 ± 2.1	1.3 ± 2.0	16.8 ± 3.2*	18.0 ± 5.5*	16.8 ± 8.9*	17.8 ± 4.7*
**Rt dPCX**	0.5 ± 2.1	0.8 ± 1.2	1.1 ± 1.4	0.4 ± 1.9	6.5 ± 2.0*	6.4 ± 1.0*	8.1 ± 5.0*	6.9 ± 2.4*
**Lt dPCX**	1.1 ± 1.2	1.3 ± 0.8	0.8 ± 1.6	1.1 ± 1.2	6.4 ± 1.2*	7.4 ± 2.2*	7.3 ± 2.5*	7.0 ± 1.7*
**Rt vPCX**	0.9 ± 2.2	0.8 ± 1.2	0.1 ± 2.0	1.1 ± 1.6	17.2 ± 7.2*^+^	16.4 ± 4.5*^+^	18.4 ± 5.6*^+§^	8.0 ± 2.0*^+ζ^
**Lt vPCX**	1.6 ± 0.9	0.5 ± 1.2	0.5 ± 2.1	0.2 ± 1.4	16.0 ± 6.2*	15.3 ± 4.1*	14.7 ± 2.0*	13.2 ± 3.0*
**Rt HP**	1.1 ± 1.6	0.8 ± 1.4	1.0 ± 0.7	0.9 ± 1.2	16.5 ± 3.4*	18.1 ± 3.9*	17.4 ± 5.9*	8.3 ± 2.1*
**Lt HP**	0.2 ± 2.1	1.0 ± 0.9	1.5 ± 1.2	1.3 ± 0.8	17.6 ± 5.7*^+^	17.6 ± 4.3*^+^	22.7 ± 8.9*^+^	15.0 ± 4.0*
**Rt TH**	0.6 ± 1.8	1.5 ± 1.2	1.0 ± 1.7	0.5 ± 0.6	10.7 ± 4.3*^+^	8.6 ± 4.4*^+^	7.3 ± 2.8*^+^	6.4 ± 2.3*
**Lt TH**	0.9 ± 1.4	0.4 ± 0.8	1.9 ± 0.8	0.1 ± 1.8	9.4 ± 4.1*	9.5 ± 5.1*	7.5 ± 2.1*	6.5 ± 2.0*
**Rt HT**	1.1 ± 2.0	1.5 ± 1.7	0.4 ± 1.6	0.8 ± 1.0	9.2 ± 4.3*	14.2 ± 6.8*	9.1 ± 4.7*	8.9 ± 4.0*
**Lt HT**	1.2 ± 1.8	1.2 ± 1.9	1.7 ± 0.6	0.5 ± 1.8	8.3 ± 3.7*	11.3 ± 4.3*	10.7 ± 2.4*	7.9 ± 4.6*
**Rt dTCX**	0.8 ± 2.4	1.2 ± 0.6	0.9 ± 1.4	0.4 ± 1.9	7.5 ± 2.8*	6.8 ± 2.7*	6.4 ± 2.4*	6.7 ± 2.7*
**Lt dTCX**	1.2 ± 1.2	0.5 ± 0.9	0.9 ± 1.5	0.1 ± 1.5	8.7 ± 3.8*	8.0 ± 2.2*	8.3 ± 2.4*	6.4 ± 3.0*
**Rt vTCX**	1.7 ± 0.9	1.1 ± 1.5	2.0 ± 0.6	0.7 ± 1.6	22.0 ± 10.5*^+^	22.8 ± 7.5*^+^	16.1 ± 5.9*^+§^	8.4 ± 1.7*^+ζ^
**Lt vTCX**	1.4 ± 1.2	1.2 ± 1.4	1.0 ± 1.1	1.0 ± 0.5	23.7 ± 10.8*	23.0 ± 13.2*	24.1 ± 10.6*	16.0 ± 3.2*
**Rt MB**	0.3 ± 2.5	1.0 ± 1.2	1.7 ± 0.8	0.7 ± 0.7	10.6 ± 2.7*	9.8 ± 3.4*	12.1 ± 4.9*	9.2 ± 3.7*
**Lt MB**	1.7 ± 1.3	1.0 ± 1.0	1.3 ± 1.1	1.7 ± 1.1	10.8 ± 4.0*	13.3 ± 4.6*	11.3 ± 4.5*	10.9 ± 3.1*
**Rt SN**	0.7 ± 2.2	0.7 ± 1.9	0.7 ± 1.5	0.3 ± 2.3	31.6 ± 7.6*	32.3 ± 4.0*	29.0 ± 9.8*^§^	11.7 ± 3.2*^ζ^
**Lt SN**	1.2 ± 2.4	0.7 ± 0.5	1.3 ± 0.8	0.8 ± 1.4	30.5 ± 6.3*	34.5 ± 7.0*	33.0 ± 14.5*	28.4 ± 10.0*
**Rt CB**	0.6 ± 1.7	0.6 ± 1.2	0.2 ± 1.6	0.0 ± 1.4	22.8 ± 6.7*^+^	22.6 ± 10.6*^+^	11.5 ± 1.9*^ζ^	10.7 ± 3.0*^ζ^
**Lt CB**	0.8 ± 1.0	1.1 ± 1.1	0.6 ± 2.1	0.7 ± 1.3	21.8 ± 4.9*^+^	22.7 ± 10.0*^+^	10.7 ± 3.3*^ζ^	11.7 ± 3.0*^ζ^
**Rt Pons**	1.5 ± 0.7	1.2 ± 1.1	0.6 ± 1.5	0.1 ± 1.9	22.3 ± 12.2*	17.5 ± 5.9*	22.7 ± 9.1*	21.7 ± 11.8*
**Lt Pons**	1.0 ± 2.4	0.9 ± 0.9	1.2 ± 0.9	0.0 ± 1.7	22.9 ± 9.8*^+^	22.7 ± 10.8*^+^	29.9 ± 10.3*^+^	17.7 ± 5.1*^+^
**SPC**	1.2 ± 1.8	0.2 ± 1.9	1.0 ± 2.8	0.3 ± 2.3	36.1 ± 5.5*	28.7 ± 13.1*	47.1 ± 6.5*^+§ζ^	85.6 ± 24.7*^+ζ^
**SPL**	0.1 ± 3.0	0.2 ± 1.5	0.8 ± 0.8	0.2 ± 1.3	10.7 ± 3.6*	13.6 ± 4.0*	21.2 ± 3.3*^+§ζ^	116.7 ± 16.6*^+ζ^
**Serum (pg/ml)**	0.0 ± 0.0	0.0 ± 0.0	0.0 ± 0.0	0.0 ± 0.0	328.8 ± 74.9*	397.2 ± 87.9*	357.4 ± 133.2*	407.8 ± 81.0*

Data represent mean±SD, *P < 0.001, compared to Veh, Kruskal-Wallis One Way Analysis of Variance on Ranks, ^+^P < 0.05, compared to corresponding Veh group, ^ζ^P < 0.05, compared to corresponding Sham group, ^§^P < 0.05, compared to corresponding female group, Kruskal-Wallis One Way Analysis of Variance on Ranks followed by Student-Newman-Keuls test for post-hoc analysis, n = 4 pups/sex/group. M: male, F: female, Rt: right, Lt: left, OB: Olfactory bulb, FC: frontal cortex, ST: striatum, dPCX: dorsal parietal cortex, vPCX: ventral parietal cortex, HP: hippocampus, TH: thalamus, HT: hypothalamus, dTCX: dorsal temporal and occipital cortex, vTCX: ventral temporal and occipital cortex, MB: midbrain, SN: substantia niagra, CB, cerebellum, SP C: cervical spinal cord, SPL: thoracic and lumbar spinal cord.

## 3 Results

### 3.1 Insulin distribution in the brain following InInsulin administration

**Fig. 1:**
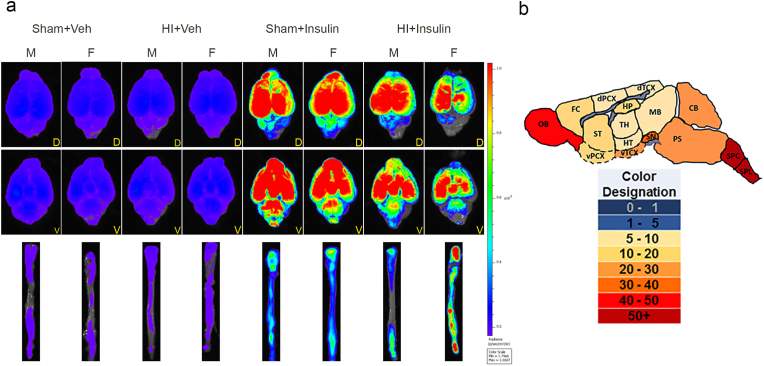
Insulin distribution in brain after InInsulin administration following HI/Sham exposure in newborn pups. (a) Ex Vivo images of dorsal (D, top raw) and ventral (V, middle raw) view of brain and spinal cord (lower raw) using live imaging devices at 15 min after intranasal administration of Alexa 546-labeled Insulin/Vehicle following prior HI/Sham at P10. Blue color indicates no to very low while red color indicates the highest insulin concentration. (b) Brain map based upon rh-insulin distribution (pg/mg of tissue) in different brain and spinal cord regions by ELISA data in male pups on right side at 15 min after InInsulin administration. M: male, F: female, OB: Olfactory bulb, FC: frontal cortex, ST: striatum, dPCX: dorsal parietal cortex, vPCX: ventral parietal cortex, HP: hippocampus, TH: thalamus, HT: hypothalamus, dTCX: dorsal temporal and occipital cortex, vTCX: ventral temporal and occipital cortex, MB: midbrain, SN: substantia nigra, CB: cerebellum, SPC: cervical spine, SPL: thoracic and lumbar spine. (For interpretation of the references to color in this figure legend, the reader is referred to the Web version of this article.)

To study the distribution of insulin in the brain following intranasal application, pups were administered Alexa-546-labeled insulin. Fig. 1a shows representative IVIS images of *ex vivo* whole-brain (dorsal and ventral view) and spinal cord 15 min after intranasal Alexa-546-insulin (red color)/Vehicle administration. The heat map clearly shows the overall bio-distribution of fluorescent labeling insulin in the central nervous system (CNS). Compared to Vehicle, both male and female pups showed higher rh-insulin on the ventral and dorsal view of the brain in the Sham + Insulin and HI + Insulin group, although images of female pups in HI + Insulin suggested relatively higher rh-insulin in the spinal cord and lower rh-insulin in brains.

To accurately assess the biodistribution of intranasal rh-insulin, another set of animals was prepared for ELISA. The concentration of rh-insulin in the different brain and spinal cord regions and serum in pups from all groups are shown in Table 1, and the brain map for insulin distribution is shown in Fig. 1b. Compared to the Vehicle group, both male and female pups in Sham + Insulin and HI + Insulin had a significantly higher rh-insulin level in serum and all brain regions on both sides of the brain (*p* < 0.05, n = 4 pups/sex/group). Compared to the corresponding sham, a very high level of rh-insulin was noted in the cervical and thoracic-lumbar spinal cord in both male and female pups in the HI + Insulin group (*p* < 0.001, n = 4 pups/sex/group). Compared to the corresponding sham, the right and left cerebellum in both sexes in HI + Insulin had a lower level (*p* < 0.05, n = 4 pups/sex/group). Compared to the corresponding sham, the right substantia nigra, ventral temporal and occipital cortex, ventral parietal cortex, striatum, frontal cortex, and left olfactory bulb in female pups from HI + Insulin had a lower level (*p* < 0.05, n = 4 pups/sex/group). Compared to females, male pups in HI + Insulin had higher insulin in the right frontal cortex, ventral parietal cortex, ventral temporal and occipital cortex, substantia nigra, and left olfactory bulb (*p* < 0.05, n = 4 pups/sex/group). Compared to females, male pups in HI + Insulin had lower insulin in the cervical and thoracic-lumbar spinal cord (*p* < 0.05, n = 4 pups/sex/group).

### 3.2 Survival rates, weights, and blood glucose

For the rest of the experiments, a total of 64 pups underwent HI, and the other 64 pups had Sham procedure, 32 of 64 pups following HI, and 32 of 64 pups following sham received InInsulin while the rest received saline. No mortality was noted in any of the four groups.

**Fig. 2:**
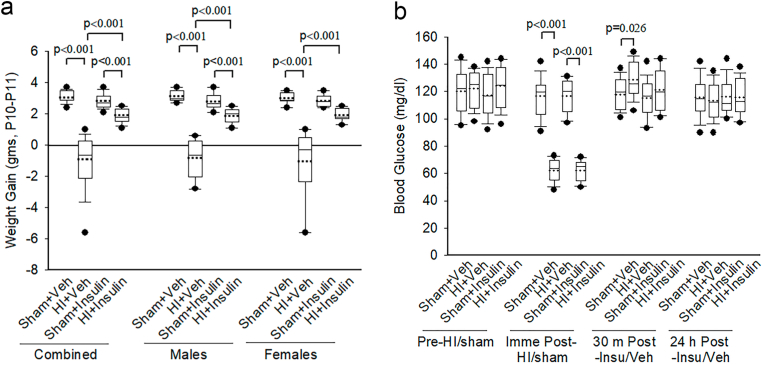
Body weight gain and blood glucose after InInsulin administration following HI/Sham exposure in neonatal pups. Rat pups were given InInsulin/Veh immediately following HI/Sham at P10. (a) Body weight gain in grams was measured at P11. Two-way ANOVA followed by Post-hoc Holm-Sidak test. Male and female pups were examined separately by the Kruskal-Wallis One Way Analysis of Variance on Ranks, followed by Post-hoc Student-Newman-Keuls test. (b) Blood glucose was measured at different time points, n = 8 pups/sex/group. Two-way ANOVA followed by Post-hoc Holm-Sidak test. Insu/Veh: Insulin/Veh. Data are presented as median and range by the box and whisker plot. Dotted line represents the mean and symbol • represents outliers.

Body weights of pups in all four groups at P10 before HI/Sham and at P11 were measured, and weight gain in each pup was calculated (Fig. 2a). The effect of Sham/HI on weight gain depended upon whether pups received InInsulin or Veh; there was a statistically significant interaction between Sham/HI and Insulin/Veh (two-way ANOVA, F (1-63) = 44.05, *p* < 0.001). On post-hoc analysis, compared to the corresponding sham, pups in HI + Insulin (*p* < 0.001) had less weight gain, but pups in HI + Veh (*p* < 0.001) had negative weight gain resulting in weight loss. Thus, weight loss in HI + Veh was much greater than HI + Insulin (*p* < 0.001, n = 16 pups/group). Sex had no interaction with the effects of HI/Sham or Insulin/Veh on weight gain (three-way ANOVA, F (1,63) = 0.0745, *p* = 0.786). When examined separately during post-hoc analysis, both male and female pups had similar findings as of all pups, except the female pups in HI + Insulin did not have statistically significant weight loss compared to the Sham + Insulin group (n = 8 pups/sex/group).

As seen in Fig. 2b, blood glucose levels were tested at various time points. Compared to the corresponding sham, pups in HI + Veh and HI + Insulin had low blood glucose immediately following HI exposure but prior to Insulin/Veh administration (*p* < 0.001, n = 16 pups/group). At 30 min after Insulin/Veh administration, pups in HI + Veh (*p* = 0.026) but not in the HI + Insulin group had statistically higher blood glucose compared to the corresponding sham. No difference in blood glucose was noted between all groups at other time points measured. Sex had no effect or no interaction with the effects of HI/Sham or Insulin/Veh on blood glucose at any time points measured.

### 3.3 InInsulin reduces HI-induced short-term neurobehavioral deficits

**Fig. 3:**
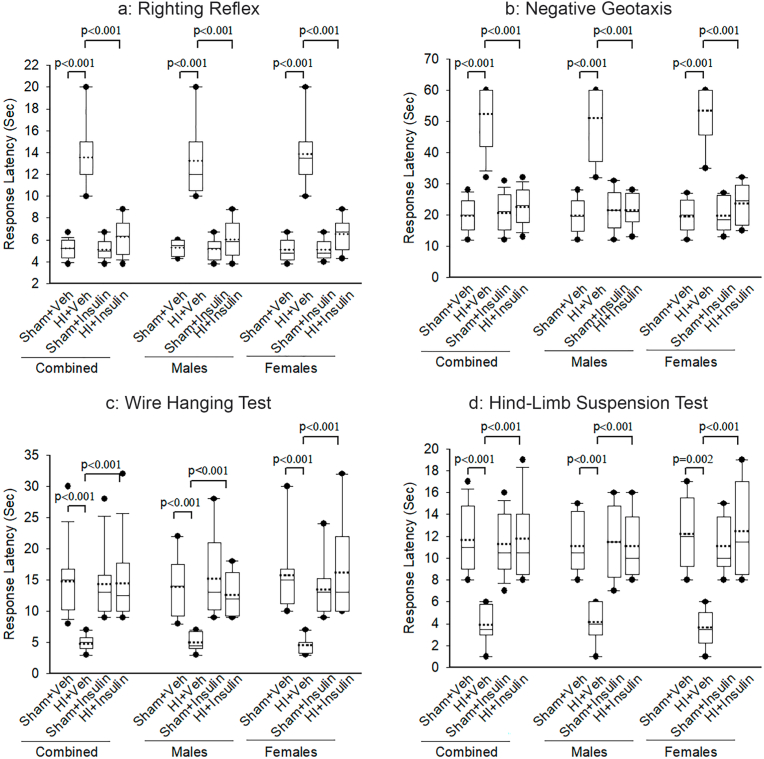
InInsulin reduces HI-induced short-term neurobehavioral deficits in newborn pups. (a) Righting reflex, (b) negative geotaxis (c) hanging wire (d) hind-limb suspension tests were performed at P11 to evaluate sensorimotor outcome following HI/Sham and immediate InInsulin/Veh treatment at P10. Two-way ANOVA followed by Post-hoc Holm-Sidak test, male and female pups were examined separately by the Kruskal-Wallis One Way Analysis of Variance on Ranks, followed by Post-hoc Student-Newman-Keuls test, n = 8 pups/sex/group. Data are presented as median and range by the box and whisker plot. Dotted line represents the mean and symbol • represents outliers.

A panel of neurobehavioral tests was performed on P11, as seen in Fig. 3. The effect of Sham/HI on the righting reflex (Fig. 3a) depended on whether pups received InInsulin or Vehicle; there was a statistically significant interaction between Sham/HI and Insulin/Veh (two-way ANOVA, F (1,63) = 58.90, *p* < 0.001). On post-hoc analysis, pups in HI + Veh took statistically significantly longer time to turn over compared to Sham + Veh (*p* < 0.001, n = 16 pups/group). Compared to HI + Veh, pups in HI + Insulin took much shorter to turn over (*p* < 0.001). There was no statistically significant difference between Sham + Insulin and HI + Insulin groups. Sex had no interaction with the effects of HI/Sham or Insulin/Veh (three-way ANOVA, F (1,63) = 0.035, *p* = 0.851). When examined separately, both male and female pups had similar findings as of analysis of male and female pups combined.

The effect of Sham/HI on the negative geotaxis test (Fig. 3b) depended on whether pups received InInsulin or Vehicle; there was a statistically significant interaction between Sham/HI and Insulin/Veh (two-way ANOVA, F (1,63) = 71.27, *p* < 0.001). On post-hoc analysis, pups in HI + Veh took a statistically significant longer time to turn and move up the incline compared to Sham + Veh (*p* < 0.001, n = 16 pups/group). Compared to HI + Veh, pups in HI + Insulin took a much shorter time (*p* < 0.001). There was no statistically significant difference between Sham + Insulin and HI + Insulin groups. Sex had no interaction with the effects of HI/Sham or Insulin/Veh (three-way ANOVA, F (1,63) = 0.028, *p* = 0.868). When examined separately, both male and female pups had similar findings as of all pups.

The effect of Sham/HI on the wire hanging test (Fig. 3c) depended on whether pups received InInsulin or Vehicle; there was a statistically significant interaction between Sham/HI and Insulin/Veh (two-way ANOVA, F (1,63) = 15.61, *p* < 0.001). On post-hoc analysis, response latency in pups in HI + Veh was statistically significant shorter compared to Sham + Veh (*p* < 0.001, n = 16 pups/group). Compared to HI + Veh, pups in HI + Insulin had a longer response latency (*p* < 0.001). There was no statistically significant difference between Sham + Insulin and HI + Insulin groups. Sex had no interaction with the effects of HI/Sham or Insulin/Veh (three-way ANOVA, F (1,63) = 2.204, *p* = 0.143). When examined separately, both male and female pups had similar findings as of all pups.

The effect of Sham/HI on the hind-limb suspension test (Fig. 3d) depended on whether pups received InInsulin or Vehicle; there was a statistically significant interaction between Sham/HI and Insulin/Veh (two-way ANOVA, F (1,63) = 33.77, *p* < 0.001). On post-hoc analysis, response latency in pups in HI + Veh was statistically significant shorter compared to Sham + Veh (*p* < 0.001, n = 16 pups/group). Compared to HI + Veh, pups in HI + Insulin had a longer response latency (*p* < 0.001). There was no statistically significant difference between Sham + Insulin and HI + Insulin groups. Sex had no interaction with the effects of HI/Sham or Insulin/Veh (three-way ANOVA, F (1,63) = 1.338, *p* = 0.252). When examined separately, both male and female pups had similar findings as of all pups.

### 3.4 InInsulin reduces HI-induced ipsilateral brain damage

**Fig. 4:**
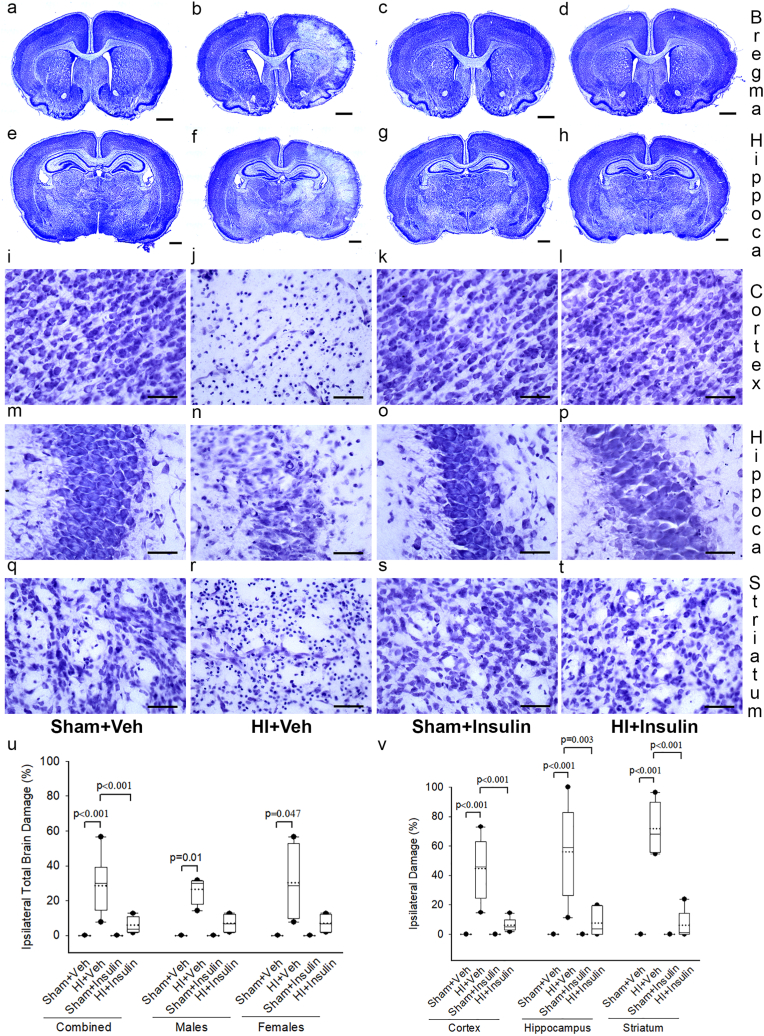
InInsulin reduces HI-induced Ipsilateral brain damage in newborn pups. Representative photomicrographs of Nissl staining evaluating brain damage at P11 following HI/Sham and immediate InInsulin/Veh treatment at P10. Coronal brain sections at the bregma (a-d) and dorsal hippocampal (e-h) levels shows obvious ipsilateral brain damage in HI + Veh groups. Scale bar = 1 mm. Representative images of cortex (i-l), hippocampus (m-p) and striatum (q-t) at higher power shows disruption of brain architecture in HI exposed pups predominantly in HI + Veh, Scale bar = 50 μm. (u) and (v) show stereology quantification of brain damage of ipsilateral hemisphere and different brain regions, respectively. Two-way ANOVA followed by Post-hoc Holm-Sidak test, male and female pups were examined separately by the Kruskal-Wallis One Way Analysis of Variance on Ranks, followed by Post-hoc Dunn test, n = 3-5 pups/sex/group. Data are presented as median and range by the box and whisker plot. Dotted line represents the mean and symbol • represents outliers.

Nissl staining of representative coronal sections of the brain at bregma and dorsal hippocampal levels is shown in Fig. 4. Subfigures a-h demonstrate Nissl staining from representative pups in all 4 groups at the bregma (a-d) and hippocampus (e-h) levels. Nissl staining showed significant damage to the ipsilateral hemisphere causing tissue loss in coronal sections of brains from pups in the HI + Veh group (b, f). Brains from pups in HI + Insulin had minimal focal damage scattered on the ipsilateral side (d, h). No brain damage was visible in both Sham groups (a, e, and c, g) and the left hemisphere in all groups (a-h). High power magnification images of the cortex (i-l), hippocampus (m-p), and striatum (q-t) from all four groups are shown. Pups from HI + Veh (j, n, r) exhibited disruption of architecture with a reduction in the number and size of cells. Pups from HI + Insulin (l, p, t) showed a minimal abnormality, while both sham groups (I, m, q, and k, o, s) showed no abnormality.

Stereology quantification of total damage on the ipsilateral side showed that the effect of Sham/HI on ipsilateral brain damage depended upon whether pups received InInsulin or Vehicle; there was a statistically significant interaction between Sham/HI and Insulin/Veh (two-way ANOVA, F (1,29) = 12.66, *p* < 0.001, Fig. 4u). On post-hoc analysis, no brain damage was noted in the sham groups, but pups in HI + Veh had, on average, 28 % total ipsilateral brain damage compared to none in Sham + Veh (*p* < 0.001, n = 6-8 pups/group). Compared to HI + Veh, pups in HI + Insulin had much less damage, on average 6% (*p* < 0.001). There was no statistically significant difference between Sham + Insulin and HI + Insulin groups. Sex had no interaction with the effects of HI/Sham or Insulin/Veh (three-way ANOVA, F (1,29) = 0.202, *p* = 0.657). When examined separately, male and female pups had similar findings; there was a trend toward reduction in brain damage in the HI + Insulin group, but it did not reach statistical significance. There was no statistically significant difference on the left side among all four groups.

Stereology quantification of regional damage of cortex (two-way ANOVA, F (1,24) = 18.75, *p* < 0.001), hippocampus (F (1,24) = 13.5, *p* = 0.001) and striatum (F (1,24) = 72.6, *p* < 0.001) on the ipsilateral side showed that the effect of Sham/HI depended upon whether pups received InInsulin or Vehicle, there was a statistically significant interaction between Sham/HI and Insulin/Veh (Fig. 4v). On post-hoc analysis, no damage was noted in the sham groups, but pups in HI + Veh had, on average, 44.5 % cortical damage, 56.2 % hippocampal damage, and 72.6% striatal damage on the ipsilateral side compared to Sham + Veh (*p* < 0.001, n = 6-7 pups/group). Compared to HI + Veh, pups in HI + Insulin had much less damage on the ipsilateral side, on average, 6.3% cortical damage, 7.6 % hippocampal damage, and 6.2% striatal damage (*p* < 0.001). There was no statistically significant difference between Sham + Insulin and HI + Insulin groups.

### 3.5 InInsulin reduces HI-induced Ipsilateral Brain Injury evaluated by immunohistochemistry

**Fig. 5:**
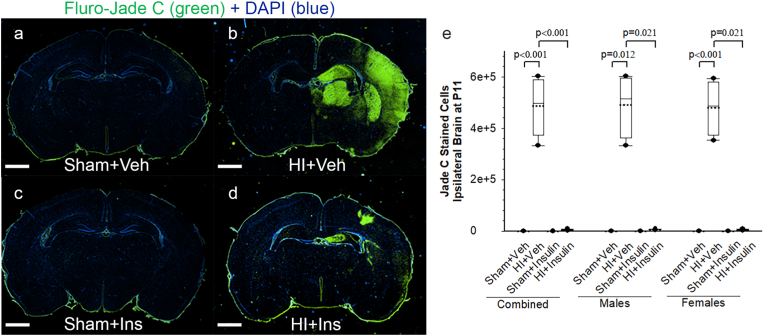
InInsulin reduces HI-induced Ipsilateral Brain Injury evaluated by Immunohistochemistry. The composite image of serial brain sections (a-d) following immunohistochemistry using Fluoro-Jade C and DAPI staining at P11 following HI/Sham and immediate InInsulin/Veh treatment at P10, Scale bar = 1 mm. Fluoro-Jade C staining identifying degenerating cells (green) and DAPI staining neuronal cells (blue) in all four groups (a-d). There was minimal to no Fluoro-Jade C + staining on the left side in all and ipsilateral side in both Sham groups. On the ipsilateral side, extensive Fluoro-Jade C + cells were seen in the HI + Veh group (b) compared to Sham + Veh (a). In contrast, fewer Jade C + cells were present in the HI + Insulin (d). Stereological quantification of the total number of Fluoro-Jade C+ cells in the cerebrum under fluorescent microscopy are presented as box and whisker graph (e). Two-way ANOVA followed by Post-hoc Holm-Sidak test, male and female pups were examined separately by the Kruskal-Wallis One Way Analysis of Variance on Ranks, followed by Post-hoc Student-Newman-Keuls test (for equal group sizes) or Dunn test (for equal group sizes), n = 4 pups/sex/group. Data are presented as median and range by the box and whisker plot. Dotted line represents the mean and symbol • represents outliers. (For interpretation of the references to color in this figure legend, the reader is referred to the Web version of this article.)

Fluoro-Jade C is widely used for the specific detection of all degenerating mature neurons, including apoptotic, necrotic, and autophagic cells. The composite image of serial brain sections (Fig. 5 a-d) following Fluoro-Jade C and DAPI staining demonstrates a lack of positively Fluoro-Jade C-stained cells in both Sham groups (a, c) and on the left side of all four groups. Strong Fluoro-Jade C positive staining was noted on the ipsilateral side in HI + Veh (b) compared to HI + Insulin (d). Stereological quantification of Fluoro-Jade C positive cells on the ipsilateral side showed that the effect of Sham/HI depended upon whether pups received InInsulin or Vehicle; there was a statistically significant interaction between Sham/HI and Insulin/Veh (two-way ANOVA, F (1,31) = 157.4, *p* < 0.001, Fig. 5e). On post-hoc analysis, no Fluoro-Jade C positive cells were noted in the sham groups, but pups in HI + Veh had significantly high Fluoro-Jade C positive cells compared to Sham + Veh (*p* < 0.001, n = 4 pups/sex/group). Compared to HI + Veh, pups in HI + Insulin had significantly fewer cells (*p* < 0.001). There was no statistically significant difference between Sham + Insulin and HI + Insulin groups. Sex had no interaction with the effects of HI/Sham or Insulin/Veh (three-way ANOVA, F (1,31) = 0.016, *p* = 0.899). When examined separately, both male and female pups had similar findings as of all pups. There was no statistically significant difference on the left side among all four groups.

### 3.6 Insulin co-localize with neuronal cells, leads to pAkt activation and subsequent reduction in HI-induced Cl-Caspase-3 positive cells in the ipsilateral brain

**Fig. 6:**
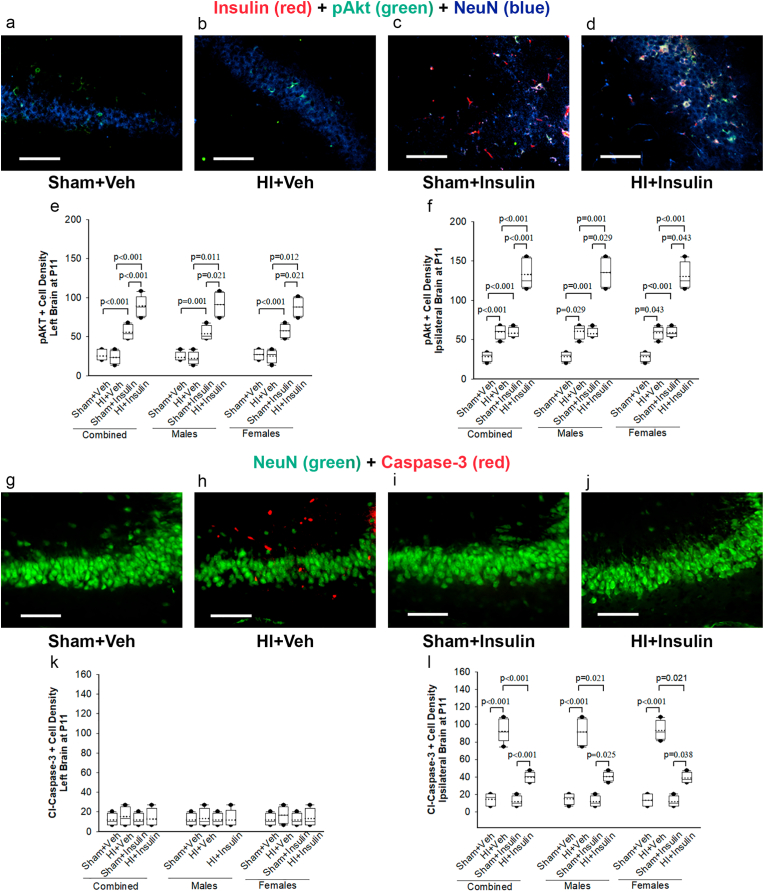
Insulin co-localizes with neuronal cells, leads to pAkt activation and subsequent reduction in HI-induced Cl-Caspase-3 positive cells in ipsilateral brain. Representative images following triple immunofluorescence (a-d) staining with Alex-546-Insulin (red), pAkt (green), and NeuN (blue) and double immunofluorescence staining (g-j) with NeuN (green) and Cl-Caspase-3 (red) of the ipsilateral hippocampal CA3 region at the dorsal hippocampal level at P11 following HI/Sham and immediate InInsulin/Veh treatment at P10 are shown, Scale bar = 100 μm. Quantification of pAkt and Cl-Caspase-3 positive cells in left (e, k) and right (f, l) hippocampal CA3 region at the dorsal hippocampal level, respectively are presented as box and whisker graphs. Two-way ANOVA followed by Post-hoc Holm-Sidak test, male and female pups were examined separately by the Kruskal-Wallis One Way Analysis of Variance on Ranks, followed by Post-hoc Student-Newman-Keuls test (for equal group sizes) or Dunn test (for equal group sizes), n = 4 pups/sex/group. Data are presented as median and range by the box and whisker plot. Dotted line represents the mean and symbol • represents outliers. (For interpretation of the references to color in this figure legend, the reader is referred to the Web version of this article.)

Immunofluorescence staining on the ipsilateral cortex revealed widespread Alex-546-Insulin-binding cells in the brain in Sham + Insulin (Fig. 6c) and HI + insulin (Fig. 6d) groups. Double-immunostaining showed that Alex-546-Insulin-bindings were primarily co-localized with NeuN, a marker for mature neuronal cells. At dorsal hippocampal levels, neuronal nuclei-positive neurons expressed pAKT in HI + Veh (Fig. 6b), Sham + Insulin (Fig. 6c), and HI + insulin (Fig. 6d) groups; expression of pAKT was much higher in HI + Insulin than HI + Veh group. Co-localization images show that compared to Sham + Insulin (Fig. 6c), higher expression of pAkt was present in the subset of Alex-546-Insulin-binding positive neuronal cells in the HI + Insulin (Fig. 6d) group. Quantification of pAkt positive cells on the ipsilateral side showed that the effect of Sham/HI depended upon whether pups received InInsulin or Vehicle; there was a statistically significant interaction between Sham/HI and Insulin/Veh (two-way ANOVA, F (1,31) = 32.4, *p* < 0.001, Fig. 6f). On post-hoc analysis, there was an increase in pAkt positive cells in the Sham + Insulin and HI + Veh groups compared to Sham + Veh (*p* < 0.001, n = 4 pups/sex/group). Compared to HI + Veh and Sham + Insulin, pups in HI + Insulin had significantly higher cells (*p* < 0.001). Sex had no interaction with the effects of HI/Sham or Insulin/Veh (three-way ANOVA, F (1,31) = 0.098, *p* = 0.757). When examined separately, both male and female pups had similar findings as of all pups. Quantification of pAkt positive cells on the left side showed that the effect of Sham/HI depended upon whether pups received InInsulin or Vehicle; there was a statistically significant interaction between Sham/HI and Insulin/Veh (two-way ANOVA, F (1,31) = 27.3, *p* < 0.001, Fig. 6e). On post-hoc analysis, there was an increase in pAkt positive cells in the Sham + Insulin compared to Sham + Veh (*p* < 0.001, n = 4 pups/sex/group). Compared to HI + Veh and Sham + Insulin, pups in HI + Insulin had significantly higher cells (*p* < 0.001). Sex had no interaction with the effects of HI/Sham or Insulin/Veh (three-way ANOVA, F (1,31) = 0.22, *p* = 0.643). When examined separately, both male and female pups had similar findings as of all pups.

Immunofluorescence staining with Cl-Caspase-3 and NeuN in the hippocampal CA3 area of the ipsilateral hemisphere at dorsal hippocampal levels (Fig. 6 g-j) showed many Cl-Caspase-3 positive cells in HI + Veh groups only; only some of them co-localized with NenN markers with a noticeable reduction in NeuN positive cells (Fig. 6h). Quantification of Cl-Caspase-3 positive cells on the ipsilateral side showed that the effect of Sham/HI depended upon whether pups received InInsulin or Vehicle; there was a statistically significant interaction between Sham/HI and Insulin/Veh (two-way ANOVA, F (1,31) = 70.6, *p* < 0.001, Fig. 6l). On post-hoc analysis, few Cl-Caspase-3 positive cells were noted in both sham groups, but pups in HI + Veh had significantly high Cl-Caspase-3 positive cells compared to Sham + Veh (*p* < 0.001, n = 4 pups/sex/group). Compared to HI + Veh, pups in HI + Insulin had significantly fewer cells (*p* < 0.001) which were still higher compared to Sham + Insulin (*p* < 0.001). Sex had no interaction with the effects of HI/Sham or Insulin/Veh (three-way ANOVA, F (1,31) = 0.158, *p* = 0.695). When examined separately, both male and female pups had similar findings as of all pups. There was no statistically significant difference on the left side among all four groups (Fig. 6k).

## 4 Discussion

The key findings of this study are that InInsulin can rapidly gain access to deep regions of the brain without causing hypoglycemia and reduces HI-induced short-term neurobehavioral deficits and ipsilateral brain damage in newborn rats. These findings support our hypothesis that InInsulin provides neuroprotection against short-term adverse outcomes following neonatal HI. Furthermore, both male and female pups show similar HI-induced short-term adverse outcomes and InInsulin-induced neuroprotection.

The newborn rat model of HI is a well-established, reproducible model and has been used routinely for pre-clinical studies by us and others (Feng et al., 2014; Hefter et al., 2018; Patel et al., 2015; Rice et al., 1981; Vannucci et al., 2005; Vannucci and Perlman, 1997). P10 rat pups are preferable to P7 rat pups used traditionally because the findings on the P7 rat brain are more closely extrapolated to an immature (32–36 weeks gestational age) human infant than a full-term infant (Patel et al., 2015). Mortality and severity of damage vary depending on the duration of hypoxia and strain of rats. In our experience, 140 min of hypoxic exposure in 7-day-old SD rat pups results in low mortality but moderate to severe brain damage (Feng et al., 2014). A variety of measures such as the pup's body temperature as well as a consistent interval (2–4 h) between arterial ligation and hypoxic exposure are required to minimize variability in the severity of brain damage in rat pups exposed to the same duration of HI exposure (Vannucci et al., 2005). In the current study, we have used 90 min of hypoxic exposure to mimic clinically more relevant mild to moderate brain injury in pups subjected to HI followed by the vehicle.

Insulin has anti-apoptotic effects (Kang et al., 2003). In an in vitro model of ischemia using mouse organotypic hippocampal slice cultures exposed to oxygen-glucose deprivation (OGD), Sun et al. show that both Insulin and Insulin-like growth factor dose-dependently reduces OGD-induced neuronal injury and Insulin-induced neuroprotection involves the PI3K/Akt pathway (Sun et al., 2010). Insulin protects neurons against excitotoxicity (Kim and Han, 2005), glutamate excitotoxicity (Kim and Han, 2005), and oxidative stress (Ribeiro et al., 2014). In short, insulin is an excellent candidate as a neuroprotective agent against neonatal HI. In human studies, insulin is detected in the cerebrospinal fluid within 30 min after nasal application (Freiherr et al., 2013), suggesting that intranasal application may be a practical means to deliver insulin into the brain parenchyma. The dose of InInsulin was extrapolated from the in vivo study of InInsulin in an adult rat model of Parkinsonism disease (Fan et al., 2019; Pang et al., 2016).

Rh-insulin ELISA and fluorescence tracing results show that the intranasal route effectively delivers human insulin to the brain in this rat model of neonatal HI. This result is similar to that of Fan et al. (2019), demonstrating the comparable insulin distribution after intranasal administration in adult male SD rats. Contrary to findings in the adult rat, a substantial concentration of insulin in the cortex is achieved in neonatal rats. The distribution of insulin in various brain regions is not directly related to that region's physical distance from the olfactory bulbs. There is a significant amount of detectable insulin in the spinal cord, particularly in the HI + Insulin groups. Currently, the mechanism of the intra-parenchymal insulin travel and distribution in the various brain regions is uncertain. Both olfactory and trigeminal nerves are considered responsible for the nose-to-brain drug transport because both innervate the nasal cavities. At the cellular level, it is proposed that either intracellular via axons of nerves or extracellular via interstitial fluid mechanism are involved (Crowe and Hsu, 2022). There is a sex-specific difference in the distribution pattern of the insulin noted in both sham/HI groups of male pups compared to the sham/HI female pups. Whether these statistical differences will translate into clinical significance is not clear.

HI causes hypoglycemia, but InInsulin does not cause hypoglycemia. There was no sex effect on these results. Insulin levels in serum following Intranasal administration in our studies were in the range of physiologic insulin levels noted in previous studies in newborn rats. These findings suggest that systemic absorption is limited following Intranasal administration. Similar to our findings, various studies in rats (Guo et al., 2017), mice (Marks et al., 2009), and humans (Benedict et al., 2004; Reger et al., 2006, 2008) all demonstrate that the intranasal insulin does not induce systemic hypoglycemia. At 30 min after Insulin/Veh administration, pups in HI + Veh do, but HI + Insulin do not have statically higher blood sugar compared to corresponding Sham. The clinical significance of this statistical difference is not clear considering that both groups’ glucose level is in the euglycemic range.

The HI insult causes decreased weight gain potential from P10 to P11 compared to sham-exposed pups; InInsulin prevents the weight loss; HI + Insulin pups demonstrated moderate weight gain. Similar weight changes secondary to HI insult have been demonstrated previously with improvements following erythropoietin (Lan et al., 2016) and minocycline (Fan et al., 2006); however, direct comparisons to this study are limited based on the differences in the age at which the insult and therapies occurred. The timings of weight loss suggest that InInsulin-induced neuroprotection prevents weight loss in the HI + Insulin group. Although, it is possible that poor weight gain following HI may contribute to the ultimate neuronal injury and repair.

InInsulin prevents the HI-induced poor performance in neurobehavioral testing in both sexes. Other investigators have examined early neuro-behavior outcomes following neonatal HI. Rat pups following 120 min of HI at P7 exhibited higher latency time in righting reflex and negative geotaxis tests as early as day 8. Abnormality in righting reflex lasted throughout the observation period of P8-P20. Pups performed better in the geotaxis test at P20 (Lubics et al., 2005). Intraperitoneal injection of erythropoietin improves but does not entirely reverse the HI-induced abnormality in righting reflex, negative geotaxis, and wire-hanging tests at P8 following HI at P7. However, the duration of hypoxia was 120 min compared to the 90 min used in our model (Lan et al., 2016).

InInsulin prevents HI-induced ipsilateral brain damage measured by Nissl and Fluoro-Jade C. Both male and female pups have similar ipsilateral brain damage as of all pups. There are regional differences in HI-induced regional volume loss, the striatum being the most vulnerable to HI than the hippocampus and the cortex. InInsulin protects the striatum, hippocampus, and cortex equally. Ours is the first report to evaluate InInsulin's neuroprotective potency in an in vivo HI brain injury model. In a neonatal pig model of HI, LeBlanc et al. used IV regular porcine insulin to prevent hyperglycemia during glucose infusion before HI injury. Piglets in the insulin group fared slightly better, but the effect was likely secondary to blood glucose levels than the direct effect of insulin in the brain (LeBlanc et al., 1997). Change et al. examined the effect of fasting hypoglycemia vs. insulin-induced hypoglycemia compared to normoglycemia in a neonatal piglet model of HI (Chang et al., 1999). The authors had achieved insulin-induced moderate hypoglycemia by a bolus injection of 1–2 μg/kg of regular insulin followed by a continuous infusion of insulin in 0.9% normal saline. The insulin flow rate was adjusted periodically to maintain the desired blood glucose level of 30–40 mg/dl. Insulin infusion was continued during HI exposure. Fasting hypoglycemia or insulin-induced hypoglycemia was not able to ameliorate brain injury. Lower brain glucose concentration was observed in the insulin-induced moderately hypoglycemic and fasting mildly hypoglycemic groups. Authors caution that insulin-induced moderate hypoglycemia might increase the risk of substrate deficiency and potential subsequent energy depletion during HI brain injury. The route, timing, and insulin dose in our study are entirely different from the previous two studies discussed, thus explaining the difference in outcomes. In models of HI brain injury in adult hyperglycemic and non-hyperglycemic rats, high insulin doses have been shown to reduce brain injury (Sanderson et al., 2009; Voll and Auer, 1991; Warner et al., 1992). The most substantial advantage of intranasal insulin treatment tested in the current project is the absence of significant hypoglycemia, even at a high dose.

The potential mechanism of the neuroprotective effect of insulin involves the PI3K pathway and subsequent activation of Akt. Insulin and the IGF-1 receptor, upon binding with their respective ligands, activate the insulin receptor substrate (IRS)/phosphatidylinositol 3-kinase (PI3K) pathway (Jiang et al., 2003; Zhang et al., 2011). PI3K phosphorylates PIP2 creating PIP3, its active form (Zhang et al., 2011). PIP3 aids in the phosphorylation of Akt into its active form, p-AKT (Jiang et al., 2003; Zhang et al., 2011; Zhou et al., 2000). Foxhead Box O transcription factors (FoxOs) regulate cellular proliferation and survival by mediating cell cycle arrest, DNA repair, and apoptosis (Zhang et al., 2011). FoxOs regulate apoptosis through the Bcl2-family of proteins, Fas ligand, and TNF-a (Zhang et al., 2011; Zhou et al., 2000). The phosphorylation of FoxOs by p-AKT downregulates the action of FoxOs, thereby downregulating pro-apoptotic mechanisms in the cell (Zhang et al., 2011; Zhou et al., 2000). P-Akt also has the downstream effect of activation of IKKα, which regulates NF-κB activation (Zhang et al., 2011). IKKα marks IκB (an inhibitor of NF-κB) for proteolysis, allowing NF-κB to aid in the transcription of pro-survival genes (Zhang et al., 2011). In an adult model of global brain ischemia, single IV bolus of 20 U/kg of insulin resulted in an early increase in pAKT after 30 min and subsequent preservation of CA1 neurons at 14 days of reperfusion (Sanderson et al., 2009). Consistent with these findings, our results show that following intranasal administration, Insulin co-localizes with neuronal cells, leading to pAkt activation and subsequent reduction in HI-induced Cl-Caspase-3 positive cells in the ipsilateral brain. These results support involvement of pAkt pathways in the InInsulin-induced neuroprotection. Although InInsulin may cause neuroprotection by another mechanisms, further studies investigating the mechanisms of InInsulin-induced neuroprotection against neonatal HI-induced brain injury are justified.

The main limitation of this study is that it has focused only on short-term neuroprotection, 24 h after the insult. Further examination is needed to determine if the neuroprotective effects of InInsulin continue to provide long-term benefits. If there is continued neuroprotection, InInsulin can be investigated as an adjuvant therapy to therapeutic hypothermia to determine if synergistic effects exist or if InInsulin allows for extending the therapeutic window for the initiation of therapeutic hypothermia. Another limitation of the study is that we did not measure pups' temperature following HI/Sham and Insulin/Veh, although pups in all groups were treated similarly to avoid any potential effects on body temperature. Additionally, in our unpublished results of another experiment in normal P10 rats, we tested the effects of different doses of InInsulin on pups’ body temperature. At any doses, including the one used in the current project, InInsulin did not change body temperature measured at 15 min, 1, and 4 h after administration. These findings rule out any possibility that InInsulin provides neuroprotection again HI by lowering body temperature. An additional limitation is the use of the rodent model of HI; caution is required in extrapolating our results from rats to human newborns.

## 5 Conclusion

In summary, InInsulin can rapidly gain access to the brain, does not cause systemic hypoglycemia, and reduces HI-induced short-term neurobehavioral deficits and ipsilateral brain damage in newborn rats. Sex does not impact the degree of short-term HI-induced brain injury or the benefit of InInsulin. If further pre-clinical research shows long-term benefits, InInsulin has the potential to be a promising non-invasive therapy to improve outcomes of newborns with HIE.

## CRediT authorship contribution statement

**Chirag P. Talati:** Conceptualization, Methodology, Investigation, Formal analysis, Writing – original draft. **Jonathan W. Lee:** Investigation. **Silu Lu:** Investigation. **Norma B. Ojeda:** Investigation, Writing – review & editing. **Varsha Prakash:** Investigation. **Nilesh Dankhara:** Investigation, Writing – review & editing. **Tanner C. Nielson:** Investigation. **Sara P. Sandifer:** Investigation. **Gene L. Bidwell:** Investigation. **Yi Pang:** Conceptualization, Investigation. **Lir-Wan Fan:** Conceptualization, Methodology, Investigation, Formal analysis, Writing – review & editing. **Abhay J. Bhatt:** Conceptualization, Methodology, Formal analysis, Writing – original draft.

## Declaration of competing interest

The authors declare the following financial interests/personal relationships which may be considered as potential competing interests: Abhay Bhatt, Lir-Wan Fan has patent #“16/891,789; Compositions, Systems, and Methods for Treating or Reducing Hypoxia-Ischemia Induced Brain Damage and Neurobehavioral Dysfunction in Neonates.” to Lir-Wan Fan, Abhay Bhatt. Pending rebuttal to examiner response.

## Data Availability

Data will be made available on request.
